# Assessing the impact of a combination of sofosbuvir and daclatasvir treatment for hepatitis C virus infection on heart rate, rhythm and heart rate variability using 24-hour ECG monitoring

**DOI:** 10.1186/s43044-020-00070-4

**Published:** 2020-07-01

**Authors:** Ahmed Mohamed El Missiri, Mona Mostafa Rayan, Mohamed Medhat Awad, Ahmed Ibrahim El Desoky

**Affiliations:** Cardiology Department, Faculty of Medicine, Ain Shams University, Abbassia square, Abbasia, Cairo, 11566 Egypt

**Keywords:** Sofosbuvir, Daclatasvir, Bradycardia, Holter, ECG monitoring

## Abstract

**Background:**

Direct-acting antiviral agents (DAAs) cure patients with hepatitis C virus (HCV) infection. Concerns have arisen the occurrence of significant bradyarrhythmias during treatment with DAAs. The aim of this study was to assess the impact of a DAA combination for the treatment of HCV infection on heart rate, rhythm, and heart rate variability (HRV) using 24-h ECG monitoring.

**Results:**

A prospective randomized study of 50 treatment-naïve patients with HCV infection treated with a combination of sofosbuvir 400 mg daily and daclatasvir 60 mg daily for 12 weeks. Surface ECG and 24-h ECG monitoring were performed at baseline and after completion of therapy to assess PR interval, corrected QT interval (QTc), minimum heart rate (HR), maximum HR, average HR, HRV time-domain and frequency-domain measures, significant pauses, tachycardias, bradycardias, premature atrial contractions (PACs), and premature ventricular contraction (PVCs).

No differences were detected in all examined parameters between baseline and after completion of treatment. PR interval was 154 ± 25.95 vs 151.4 ± 23.82 ms, respectively (*p* = 0.124). QTc interval was 397.34 ± 29.38 vs 395.04 ± 30.23 ms, respectively (*p* = 0.403). No differences were detected for minimum HR, maximum HR, average HR, HRV time-domain and frequency-domain measures, the occurrence of significant pauses, sinus tachycardia episodes, sinus bradycardia episodes, PACs, and PVCs. No episodes of bradyarrhythmias, syncope, and atrial fibrillation, supraventricular, or ventricular tachycardias were reported or detected.

**Conclusion:**

In non-cardiac patients receiving no cardioactive medications, the combination of sofosbuvir and daclatasvir for the treatment of HCV infection has no effect on HR, rhythm, conductivity, or HRV. No symptomatic bradycardias, tachycardias, or syncope were reported or detected using 24-h ECG monitoring.

## Background

Infection with hepatitis C virus (HCV), a hepatotropic RNA virus, causes progressive liver damage that may lead to liver cirrhosis and hepatocellular carcinoma. It is the leading cause of chronic liver disease in several countries with up to 100 million people chronically infected. Nearly 50% of those infected with HCV are in China, Pakistan, India, Egypt, and Russia. HCV infection produces a range of effects on the liver from minimal histological changes to extensive fibrosis and cirrhosis where the liver architecture becomes distorted and regenerative nodules are formed [1, 2].

For a long period, interferon (IFN)-based therapies were the mainstay; however, they were later abandoned due to the lack of drug tolerability and the presence of many adverse events. The need arose for a new class of medications to treat HCV infections which was tolerable and had fewer adverse events. This new class is the so-called direct-acting antiviral agents (DAA). These agents have a high cure rate for patients with HCV infection and are particularly useful for previously difficult cases such as those having liver decompensation, co-infection with human immunodeficiency virus (HIV), and those with renal impairment [3, 4].

DAAs, also called novel IFN-free therapeutic agents, gained breakthrough therapy status by the US Food and Drug Administration. However, retrospective studies, case reports, and post-marketing reports suggest that DAAs might cause cardiac adverse events [5]. Treatment with sofosbuvir, one of the DAAs, was generally associated with a rate of less than 5% of serious adverse events, but significant bradyarrhythmias were reported especially when such DAAs were co-administered with amiodarone and possibly other negative chronotropic drugs commonly used in cardiac patients [6, 7].

The mechanism causing such adverse events during treatment with sofosbuvir is not clear and the manufacturer reported in 2015 that the number of patients having significant bradyarrhythmias requiring pacing was very small and most had co-administration of amiodarone. Manufacturer recommendations at that time were to review the medications of patients, consider risk factors for bradyarrhythmias, and monitor cardiac rhythm during the initiation of therapy with sofosbuvir [6, 8].

The aim of this study was to assess the impact of the DAAs combination sofosbuvir and daclatasvir as part of the national regimen of HCV infection treatment on heart rate, rhythm, and heart rate variability (HRV) using 24-h ECG monitoring.

## Methods

The study protocol was approved by the institutional ethical committee. Written informed consents were obtained from all participants prior to enrollment in this study.

This was a prospective randomized study performed on 50 treatment-naïve adult patients with chronic HCV infection—confirmed by a polymerase chain reaction (PCR) test to have HCV genotype 4—presenting to the specialized virology clinic at our institution from February 2019 to August 2019.

All patients were treated according to the national protocol for the treatment of chronic HCV infection which is in line with the recommendations of the European Association for the Study of the Liver [9]. Patients received sofosbuvir 400 mg daily and daclatasvir 60 mg daily for 12 weeks.

Patients were excluded from the study if they had any of the following criteria: Age less than 18 years old; co-administration of beta blockers, calcium channel blockers, amiodarone, or ivabradine; history of coronary artery disease; history of heart failure; more than mild valvular stenosis or more than moderate valvular regurgitation; atrial fibrillation; history of arrhythmias; uncontrolled hypertension; co-infection with hepatitis B virus, HIV, or bilharziasis; an estimated glomerular filtration rate less than 30 ml/min; previous treatment for HCV infection; current liver cirrhosis; anemia; thyrotoxicosis; or active inflammation.

### Patient interviews

Patients were interviewed for a detailed history and physical examination. Presence of any of the exclusion criteria was assessed. General and local abdominal examination aimed to detect manifestations of chronic liver diseases and exclude liver cirrhosis. Local cardiac examination aimed to exclude undiagnosed cardiac conditions.

Venous blood samples were obtained from all patients to perform quantitative PCR of HCV antibody prior to treatment and following the completion of treatment, in addition, to routine baseline investigations which are a prerequisite for patient selection for suitability for DAA treatment according to the national protocol. These include aspartate transaminase, alanine transaminase, total bilirubin, serum albumin, alkaline phosphatase, prothrombin time, international normalized ratio, serum creatinine, glycated hemoglobin, complete blood count, and alpha fetoprotein. Additionally, abdominal ultrasound was performed to detect the presence of liver cirrhosis, splenomegaly, or ascites.

### Cardiac investigations

The following was performed before the start of treatment and after completion of the 12-week regimen (for practical purposes, the follow-up cardiac investigations were performed during any of the last 3 days of the treatment regimen):

*Surface ECG*: to assess heart rate, rhythm, measure PR interval, and estimate the corrected QT interval duration (QTc).

PR interval duration was measured from the beginning of the P wave to the beginning of the QRS complex. QT interval was measured from the beginning of the QRS complex to the end of the downward slope of the T wave crossing of the isoelectric line, and then, QTc was calculated according to the formula QTc = QT/square root of R–R interval [10, 11].

*24-h ECG monitoring*: This was performed using a NORAV Medical NR-302 device which is a three-channel Holter monitor (NORAV Medical GmbH, Wiesbaden, Germany) with five ECG cables connected in designated locations on each patient’s chest.

Device attachment: to ensure a proper recording with minimal artifacts, all patients were prepared by hair shaving (if present) at the site of electrode attachment; the skin was cleaned and gently abraded to improve conductivity and to hold the electrodes in place for the whole duration of the recording. Cables were then connected to the electrodes at the pre-defined sites, and the required identifier data was input to the device.

Device data analysis: all recordings were analyzed by an experienced electrophysiologist blinded to the patient information. The following variables were assessed: (1) minimum heart rate; (2) maximum heart rate; (3) average heart rate; (4) HRV time-domain and frequency-domain measures; (5) significant pauses (longer than 2.5 s); (6) sinus tachycardia episodes (where sinus heart rate exceeds 100 bpm); (7) sinus bradycardia episodes (where sinus heart rate is less than 60 bpm); (8) number of premature atrial contractions (PACs); (9) number of premature ventricular contraction (PVCs); (10) episodes of atrial fibrillation, supraventricular, or ventricular tachycardias.

#### *HRV time-domain measures*:

The following time-domain measures were assessed to estimate the variability in the measurements of the time period between successive heartbeats [12–14]:

- SDNN which is the standard deviation of NN intervals measured in milliseconds.

- SDANN which is the standard deviation of the average NN intervals for each 5 min segment of the 24-h HRV recording measured in milliseconds.

- RMSSD which is the root mean square of successive RR interval differences measured in milliseconds.

- HRV triangular index which is the integral of the density of the RR interval histogram divided by its height.

#### *HRV frequency-domain measures*:

Frequency-domain measures were assessed to estimate the distribution of power in four frequency bands where power is the signal energy found within a frequency band. The European Society of Cardiology and the North American Society of Pacing and Electrophysiology task force divided heart rate oscillations into ultra-low-frequency (ULF), very-low-frequency (VLF), low-frequency (LF), and high-frequency (HF) bands. The following measures were assessed [12–14]:

- ULF power which is the absolute power of the ULF band (≤ 0.003 Hz).

- VLF power which is the absolute power of the VLF band (0.003–0.04 Hz).

- LF power which is the absolute power of the LF band (0.04–0.15 Hz).

- HF power which is the absolute power of the HF band (0.15–0.4 Hz).

where absolute power for the previous measures is calculated in milliseconds squared divided by cycles per second [12–14].

### Statistics

Data from the 50 patients were collected, coded, and statistically analyzed using IBM Statistical Package for Social Science (IBM Corporation, Armonk, NY, USA). Continuous variables that passed normality test were expressed as mean ± standard deviation and analyzed using two-tailed Student’s *t* test. Categorical variables were expressed as number and percentage and analyzed using chi-squared test. The level of significance was defined at a *p* value less than 0.05. All baseline parameters were statistically homogenous and normally distributed giving a study power of more than 80.

## Results

### Baseline patient characteristics

Mean patient’s age was 48.32 ± 16.4 years old. Females represented 36% (*n* = 18). Twenty-six percent (*n* = 13) were diabetic and 44% (*n* = 22) were hypertensive.

### Twelve-lead surface ECG

There was no difference in PR interval duration on the surface ECG between baseline and after completion of treatment 154 ± 25.95 vs 151.4 ± 23.82 ms (*p* = 0.124).

There was no difference in QTc interval duration on the surface ECG between baseline and after completion of treatment 397.34 ± 29.38 vs 395.04 ± 30.23 ms (*p* = 0.403) (Table 1).

**Table 1:** Comparing surface ECG and Holter monitoring variables at baseline and after completion of treatment

Variable	Baseline (*n* = 50)	After completion of treatment (*n* = 50)	*p* value
**Surface ECG**			
PR interval, ms	154 ± 25.95	151.4 ± 23.82	0.124
QTc interval, ms	397.34 ± 29.38	395.04 ± 30.23	0.403
**24-h ECG (Holter) monitoring**			
Minimum heart rate, bpm	55.88 ± 9.2	56.66 ± 9.45	0.457
Maximum heart rate, bpm	129.08 ± 20.07	128.98 ±16.89	0.964
Average heart rate, bpm	79.44 ±10.14	79.96 ± 8.77	0.534
Patients with significant pauses, *n* (%)	0 (0%)	0 (0%)	1
Patients with sinus tachycardia episodes, *n* (%)	47 (94%)	47 (94%)	1
Patients with sinus bradycardia episodes, *n* (%)	35 (70%)	33 (66%)	0.83
Patients with PACs, *n* (%)	6 (12%)	3 (6%)	0.485
Patients with PVCs, *n* (%)	22 (44%)	14 (28%)	0.145

Continuous variables are expressed as mean and standard deviation, whereas categorical variables are expressed as number (percentage)

*QTc interval* corrected QT interval, *PACs* premature atrial contraction, *PVCs* premature ventricular contractions

### Twenty-four-hour ECG monitoring

No difference was detected on comparing parameters at baseline and after completion of treatment for all assessed variables (minimum heart rate, maximum heart rate, average heart rate), as well as the number of patients with significant pauses, sinus tachycardia episodes, sinus bradycardia episodes, PACs, and PVCs as detailed in Table 1.

No episodes of bradyarrhythmias, syncope, and atrial fibrillation, supraventricular, or ventricular tachycardias were reported or detected by monitoring.

### HRV measures

No difference was detected on comparing HRV time-domain and frequency-domain measures at baseline and after completion of treatment as detailed in Table 2.

**Table 2:** Comparing heart rate variability (HRV) time-domain and frequency-domain measures at baseline and after completion of treatment

Variable	Baseline (*n* = 50)	After completion of treatment (*n* = 50)	*p* value
**HRV time-domain measures**			
SDNN, ms	121.70 ± 24.24	133.56 ± 50.79	0.122
SDANN, ms	111.99 ± 31.13	113.03 ± 79.76	0.913
RMSSD, ms	36.68 ± 19.49	105.06 ± 118.48	0.21
HRV triangular index	37.39 ± 16.69	27.21 ± 12.15	0.31
**HRV frequency-domain measures**			
ULF power, ms^2^	11308 ± 4001.5	7270.4 ± 4551.4	0.092
VLF power, ms^2^	1566.5 ± 1454.4	1331.1 ± 865.41	0.876
LF power, ms^2^	1103.2 ± 1290.6	2584.1 ± 4392.2	0.225
HF power, ms^2^	709.28 ± 724.54	4323.2 ± 8251.2	0.165
LF/HF ratio	2.85 ± 3.62	1.39 ± 0.846	0.194

Continuous variables are expressed as mean and standard deviation

*HRV* means heart rate variability; *SDNN* means standard deviation of NN intervals; *SDANN* means standard deviation of the average NN intervals for each 5 min segment of a 24-h HRV recording; *RMSSD* means root mean square of successive RR interval differences; *ULF power* means absolute power of the ultra-low-frequency band (≤ 0.003 Hz); *VLF power* means absolute power of the very-low-frequency band (0.0033–0.04 Hz); *LF power* means absolute power of the low-frequency band (0.04–0.15 Hz); *HF power* means absolute power of the high-frequency band (0.15–0.4 Hz)

### Comparing patients with electrical events

After excluding patients with no electrical events for each of the parameters tested, there was no difference in the average number of sinus tachycardia episodes, sinus bradycardia episodes, PACs, and PVCs at baseline compared to after completion of treatment as shown in Table 3.

**Table 3:** Comparing patients with electrical events on Holter monitoring at baseline and after completion of treatment

Variable	Baseline	After completion of treatment	*p* value
Sinus tachycardia episodes, *n* = 48	35.29 ± 46	29.69 ± 27.15	0.81
Sinus bradycardia episodes, *n* = 40	7.88 ± 7.56	7.95 ± 8.1	0.795
PACs, *n* = 8	6.63 ± 7.37	7.5 ± 17.3	0.89
PVCs, *n* = 27	71.6 ± 147	31.89 ± 69.26	0.124

Continuous variables are expressed as mean and standard deviation. PA Cs premature atrial contractions PVCs premature ventricular contractions

## Discussion

HCV infection is one of the leading causes of liver fibrosis, cirrhosis, and hepatocellular carcinoma. It is a major health problem and one of the leading causes of chronic liver disease worldwide. The introduction of DAAs, which target the life cycle of HCV at different stages, represented a major shift in the treatment of such patients with very high cure rates. A few case series and post-marketing reports suggested possible effects of DAAs on cardiac rhythm where significant bradycardias were reported which required pacing in a limited number of patients [2, 4, 6, 15]. In one of those case series, three patients required permanent pacing due to non-resolving junctional escape rhythm with a ventricular rate of 30 bpm in the first, recurrent sinus bradycardia with syncope in the second, and intermittent third-degree atrioventricular block with syncope in the third patient [6].

This study aimed to assess the impact of DAAs combination used in the national regimen of HCV infection treatment on heart rate, rhythm, and heart rate variability using 24-h ECG monitoring. The need for this study arose from the frequent consultations that cardiologists and internists meet in countries were HCV is endemic regarding the hepatologist’s “fear” of occurrence of bradyarrhythmias. To the best of our knowledge, this is the first study to use 24-h ECG monitoring to detect arrhythmias and HRV in HCV-infected patients treated with DAAs.

The main findings of this study are that the combination of the DAAs sofosbuvir and daclatasvir for the treatment of HCV infection does not affect heart rate (minimum, maximum, and average) and heart rhythm (frequency of sinus tachycardia episodes, sinus bradycardia episodes, PACs, PVCs, occurrence of atrial fibrillation, supraventricular, or ventricular tachycardias) detected using 24-h ECG monitoring. Additionally, this combination had no effect on HRV time-domain and frequency-domain measures. No symptomatic tachyarrhythmias, bradyarrhythmias, or syncope were reported in all patients during the treatment period.

A study was performed to assess the cardiovascular effects of DAAs in patients with HCV infection. It included 170 patients with HCV infection: 100 patients received a triple combination therapy of pegylated IFN alfa, sofosbuvir, and ribavirin, while 70 patients received a dual combination therapy of sofosbuvir and simeprevir. No arrhythmias or changes in QTc interval were observed in any patients before and after therapy using the 12-lead surface ECG [16].

A study in 2015 examined the cardiotoxic effects associated with the use of a DAA called “BMS-986094” in the treatment of HCV infection that had been prematurely discontinued. This study involved 34 patients, 14 of which experienced rapidly progressive heart failure and 11 required hospitalization for suspected cardiotoxicity. The following ECG abnormalities were detected: three cases developed intraventricular conduction defect, two cases developed sinus bradycardia, and one case developed borderline prolongation of QTc interval [17].

A meta-analysis and systemic review examining 2346 patients from six trials for the cardiac safety of sofosbuvir in the treatment of HCV infection found that sofosbuvir was not associated with an increased risk of cardiac outcomes, including arrhythmias and bradycardias [18].

Another study was performed on 39 patients with HCV infection treated with DAAs, 26 of which treated with sofosbuvir, to assess ECG changes. A mild, transient increase in QTc occurred after 1 week from 424.3 to 431.2 ms (*p* = 0.013) that returned to baseline at 2 weeks and remained normal till the end of treatment. Such changes were not clinically significant, and no cases of symptomatic bradycardia or syncope were reported. All patients in this study did not receive amiodarone or other medications affecting heart rate [19].

Another study aimed to examine the cardiac safety of using a combination of sofosbuvir (400 mg) and ledipasvir (90 mg) in a single oral tablet daily for 12 weeks in 40 children and adolescents (12–17 years old) with HCV infection. Twelve-lead surface ECG was performed at baseline and after 4 and 12 weeks of therapy. No cardiac symptoms were reported during treatment, and there were no cases of symptomatic bradycardia or syncope. It was concluded that treatment with sofosbuvir and ledipasvir combination did not cause any bradyarrhythmias during treatment [20].

On the other hand, the co-administration of amiodarone with the DAAs sofosbuvir and daclatasvir was reported in two cases to cause significant bradycardia. One case developed asystole 30 minutes after the administration of sofosbuvir and daclatasvir. Amiodarone, sofosbuvir, and daclatasvir treatment were consequently stopped. Ten days later, cardiac evaluation was normal, and the patient was discharged. Another case taking amiodarone and propranolol had extreme sinus node dysfunction with a heart rate of 27 bpm 2 h after the administration of sofosbuvir and daclatasvir. Amiodarone and propranolol were stopped; however, the patient continued sofosbuvir and daclatasvir for 3 days and sinus bradycardia was detected each day 2 h after intake of these drugs. When sofosbuvir and daclatasvir were stopped, no bradycardia was observed. Additionally, administration of sofosbuvir and daclatasvir on day 13 induced bradycardia 2 h after their intake. However, no bradycardia occurred following a re-challenge 8 weeks after the patient had stopped taking amiodarone. The authors indicated that it appears that patients treated with amiodarone should be continuously monitored within the first 48 h following the initiation of sofosbuvir and daclatasvir [21].

Another case reported frequent PVCs within the early hours after administration of sofosbuvir in a patient treated with propranolol. The PVCs disappeared within 24 h after stopping sofosbuvir [22].

To the best of our knowledge, no other studies examined the effect of DAAs on HRV measures. However, the effect of the antiviral IFN-alpha on HRV was examined in a few studies. One of them examined 11 patients with chronic active hepatitis (10 had chronic hepatitis C and one had chronic hepatitis B) for the effect of treatment with IFN-alpha on HRV measures of LF power, HF power, and LF/HF ratio. The authors concluded that IFN-alpha therapy did not cause any changes in the power spectrum of R–R intervals and thus did not change either sympathetic or parasympathetic outflow and heart rate variability [23].

Another study on 22 HCV patients treated with a combination of IFN-alpha and ribavirin examined for changes in HRV at baseline and after 12, 24, and 48 weeks of antiviral therapy. Authors reported an early rise in autonomic dysfunction (indicated by decreased HRV time and frequency-domain indices after 12 weeks) that was fully reversible at week 48 of therapy [24].

On the other hand, a study performed on nine patients with chronic hepatitis C with no history of cardiac disease before and after treatment with IFN-alpha examined the HRV parameters SDNN, SDANN, RMSSD, and pNN50, in addition to the frequency analysis of HR in LF and HF. The authors reported a decrease in RMSSD and pNN50 measures of HRV. They concluded that the arrhythmogenic effect of INF-alpha may be caused by decreased HRV, and they recommended that INF-alpha therapy should be contraindicated in patients predisposed to severe cardiac disorders, especially arrhythmias, ischemia, and heart failure [25].

## Study limitations

The limitations of this study are that it comes from a single medical center with a relatively small number of patients. Concomitant use of negative chronotropic drugs was not examined. Patients with cardiac diseases were excluded from the study.

## Conclusion

In non-cardiac patients receiving no cardioactive medications, the combination of sofosbuvir and daclatasvir for the treatment of HCV infection has no effect on heart rate, rhythm, conductivity, or heart rate variability. No symptomatic bradycardias, tachycardias, or syncope were reported or detected using 24-h ECG monitoring during treatment.

## Data Availability

The datasets used and analyzed during the current study are available from the corresponding author on reasonable request.

## Abbreviations

HCV: Hepatitis C virus
DAA: Direct-acting antiviral agents
ECC: Electrocardiogram
QTc: Corrected QT interval
HR: Heart rate
HRV: Heart rate variability
PAC: Premature atrial contraction
PVC: Premature ventricular contraction
IFN: Interferon
HIV: Human immunodeficiency virus
PCR: Polymerase chain reaction
SDNN: Standard deviation of NN intervals
SDANN: Standard deviation of the average NN intervals
RMSSD: Root mean square of successive RR interval differences
ULF: Ultra-low-frequency band
VLF: Very-low-frequency band
LF: Low-frequency band
HF: High-frequency band
